# Different immunological mechanisms between AQP4 antibody-positive and MOG antibody-positive optic neuritis based on RNA sequencing analysis of whole blood

**DOI:** 10.3389/fimmu.2023.1095966

**Published:** 2023-03-09

**Authors:** Xuelian Chen, Libo Cheng, Ying Pan, Peng Chen, Yidan Luo, Shiyi Li, Wenjun Zou, Ke Wang

**Affiliations:** ^1^Department of Ophthalmology, Affiliated Wuxi Clinical College of Nantong University, Wuxi, Jiangsu, China; ^2^Department of Ophthalmology, Jiangnan University Medical Center (JUMC), Wuxi, Jiangsu, China; ^3^Department of Ophthalmology, Wuxi No.2 People’s Hospital, Wuxi, Jiangsu, China; ^4^Department of Ophthalmology, The Affiliated Wuxi No.2 People’s Hospital of Nanjing Medical University, Wuxi, Jiangsu, China; ^5^National Health Commission (NHC) Key Laboratory of Nuclear Medicine, Jiangsu Key Laboratory of Molecular Nuclear Medicine, Jiangsu Institute of Nuclear Medicine, Wuxi, Jiangsu, China; ^6^Department of Radiopharmaceuticals, School of Pharmacy, Nanjing Medical University, Nanjing, Jiangsu, China

**Keywords:** optic neuritis, aquaporin 4, myelin oligodendrocyte glycoprotein, RNA-Seq, toll-like receptors

## Abstract

**Purpose:**

To compare the different immunological mechanisms between aquaporin 4 antibody-associated optic neuritis (AQP4-ON) and myelin oligodendrocyte glycoprotein antibody-associated optic neuritis (MOG-ON) based on RNA sequencing (RNA-seq) of whole blood.

**Methods:**

Whole blood was collected from seven healthy volunteers, 6 patients with AQP4-ON and 8 patients with MOG-ON, and used for RNA-seq analysis. An examination of immune cell infiltration was performed using the CIBERSORTx algorithm to identify infiltrated immune cells.

**Results:**

RNA-seq analysis showed that the inflammatory signaling was mainly activated by *TLR2*, *TLR5*, *TLR8* and *TLR10* in AQP4-ON patients, while which was mainly activated by *TLR1*, *TLR2*, *TLR4*, *TLR5* and *TLR8* in MOG-ON patients. Biological function identification of differentially expressed genes (DEGs) based on Gene Ontology (GO) term and Kyoto encyclopedia of genes and genomes (KEGG) pathway enrichment analysis, as well as Disease Ontology (DO) analysis, showed that the inflammation in AQP4-ON was likely mediated by damage-associated molecular pattern (DAMP), while which in MOG-ON was likely mediated by pathogen-associated molecular pattern (PAMP). Analysis of immune cell infiltration showed that the proportion of immune cell infiltration was related to patients’ vision. The infiltration ratios of monocytes (rs=0.69, *P*=0.006) and M0 macrophages (rs=0.66, *P*=0.01) were positively correlated with the BCVA (LogMAR), and the infiltration ratio of neutrophils was negatively correlated with the BCVA (LogMAR) (rs=0.65, *P*=0.01).

**Conclusion:**

This study reveals different immunological mechanisms between AQP4-ON and MOG-ON based on transcriptomics analysis of patients’ whole blood, which may expand the current knowledge regarding optic neuritis.

## 1 Introduction

Optic neuritis (ON) is an inflammatory demyelinating disease that mainly affects young people, and its prevalence rate is about 1 to 6 per 100,000 population (1). In patients with non-infectious ON, 12% were aquaporin 4-immunoglobulin G (AQP4-IgG) positive and 10% were myelin oligodendrocyte glycoprotein-IgG (MOG-IgG) positive (2). Patients with AQP4-IgG related ON (AQP4-ON) have worse vision and usually develop blindness, while patients with MOG-ON have a higher recurrence rate (3, 4).

AQP4, the target protein attacked by AQP4-IgG, is responsible for maintaining water homeostasis and solute transfer, and can provide a fast water transport channel for astrocytes (5). Meanwhile, MOG, the target antigen recognized by MOG-IgG, is involved in myelin sheath adhesion and maintaining the integrity of the myelin sheath (6). Due to the distinct functions of AQP4 and MOG, the clinical features and pathological lesions caused by AQP4-IgG and MOG-IgG are different (7, 8). Besides, the co-existence of MOG-IgG and AQP4-IgG is highly uncommon (rate of 0.06%), indicating that AQP4-ON and MOG-ON may be developed through different immunological mechanisms (9, 10). However, to date, there have been no reports on the difference between the two types of immune-mediated ON.

We investigated the molecular mechanisms and therapeutic targets of AQP4-ON and MOG-ON by bioinformatic analysis. Whole blood samples from patients with AQP4-ON and MOG-ON as well as healthy control (HC) subjects were subjected to RNA sequencing (RNA-seq) analysis. The key differentially expressed genes (DEGs) in AQP4-ON and MOG-ON patients were comprehensively analyzed, and on this basis, the immune cell infiltration rates in all patients were analyzed.

## 2 Materials and methods

### 2.1 Subjects and samples

All subjects were recruited at the Affiliated Wuxi Clinical College of Nantong University from November 2020 to August 2022. The diagnosis of ON was made following the guidelines of the Optic Neuritis Treatment Trial (ONTT) study (11). The inclusion criteria were (1): serum AQP4-IgG or MOG-IgG was positive (2), the first attack of ON and in the stage of acute attack and (3) no treatment with glucocorticoid and other drugs. Exclusion criteria were (1) neurological disease such as encephalitis and myelitis (2), other eye diseases except cataract, such as glaucoma、uveitis and ischemic optic neuropathy. For high sensitivity and absolute specificity, the AQP4-IgG was measured using an enzyme-linked immunosorbent assay (ELISA) kit (RSR Ltd., Cardiff, UK) according to the previous study (12) and results 3.0 u/mL were considered as positive. The MOG-IgG was measured by indirect immunofluorescence testing (IIFT) using a cell-based assay (CBA), and the normal value was negative. For statistical analysis, the Snellen best-corrected visual acuity (BCVA) was transformed to logarithm of minimum angle of resolution (LogMAR) units (13). The ethics committee of the Affiliated Wuxi Clinical College of Nantong University approved the use of blood samples for scientific research purposes (certificate No.2021 Y-33), and all participants signed written informed consent.

### 2.2 RNA extraction and library preparation

Peripheral venous whole blood was collected from patients and HC subjects in vacuum tubes containing EDTA as an anticoagulant. After blood collection, 4 mL of TRIzol reagent (Beyotime, Nantong, China) was added to each blood sample and stored at −80°C for subsequent RNA extraction. Subsequently, after collecting all the samples, total RNA was extracted from each sample using the TRIzol extraction method, following the manufacturer’s instructions. After verifying its integrity, the total RNA was reverse transcribed into cDNA, and then the cDNA was size selected to 250-300 bp using AMPure XP beads (Beckman Coulter Inc., Brea, CA, USA). Following polymerase chain reaction (PCR) amplification, the PCR product was once more purified using AMPure XP beads to prepare the cDNA library. The quality of the library was assessed using the Agilent 2100 bioanalyzer (Agilent Technologies Inc., Santa Clara, CA, USA), and then sequenced using the Illumina NovaSeq 6000 platform (Illumina Inc., San Diego, CA, USA).

### 2.3 Data quality control and quantification

Cleaned data were obtained by filtering the original data, and the cleaned reads were compared with the reference genome using the HISAT2 v2.0.5 software (http://daehwankimlab.github.io/hisat2/). The new genes were predicted by the StringTie (1.3.3b) software, and the gene expression level was quantified by feature counts (1.5.0-P3). The raw data have been submitted to GEO database (GEO accession numbers: GSE217410).

### 2.4 DEGs analysis

The DESeq (1.20.0) software was used to analyze the DEGs (relative to HC group) between the two groups of patients (http://www.bioinformatics.babraham.ac.uk/pro-jects/trim_galore/). The Benjamini and Hochberg method was used to adjust the *P* value and the false discovery rate for multiple comparisons. The genes with *P* value ≤ 0.05 adjusted by DESeq2 were considered to be differentially expressed.

### 2.5 Enrichment analysis of DEGs

Gene Ontology (GO) enrichment analysis (as implemented in the cluster Profiler R package (3.8.1)), was performed on DEGs, in which gene length bias was corrected. The ClusterProfiler (3.8.1) software package in the R platform was used to perform the Kyoto encyclopedia of genes and genomes (KEGG) pathway enrichment analysis of DEGs. The Disease Ontology (DO) is a knowledge database related to human diseases that describes the association of human genes to diseases.

### 2.6 Protein-protein interaction network analysis

The STRING software was used to predict the differential protein-protein interaction (PPI) network from the interaction relationship in the STRING protein interaction database, and then Cytoscape V3.9.1 was used to visually edit it to find the module network and prioritize the genes (14).

### 2.7 Immunocyte infiltration analysis

Cell-type identification by estimating relative subsets of RNA transcripts (CIBERSORTx) (https://cibersortx.stanford.edu) is a method to characterize the cell composition of complex tissues from gene expression profiles (GEPs). The CIBERSORTx algorithm was used to analyze 22 immune cell subsets significantly differentially expressed in all patients and control subjects. Then, the Immune Cell Abundance Identifier (ImmuCellAI) gene set signature-based method (http://bioinfo.life.hust.edu.cn/ImmuCellAI#!/) was used to estimate the proportion of infiltrated immune cell types.

### 2.8 Statistical analysis

Statistical analysis was performed using the IBM SPSS statistical 26.0. software for macOS (IBM Corporation, Armonk, NY, USA). For statistics, Snellen visual acuity was converted to logarithm of the minimum angle of resolution (logMAR). Counting fingers vision was converted to a value of 2.0 logMAR, hand motion vision was converted to a value of 2.5 logMAR, light perception vision was converted to a value of 3.0 logMAR and no light perception vision was converted to a value of 3.5 logMAR (15). Fisher’s exact test was used for comparison of clinical characteristics among the groups. One-way analysis of variance (ANOVA) was used to compare normally distributed data, and the Kruskal-Wallis test was used for non-normally distributed data. Spearman correlation analysis was used to evaluate the relationship between data and vision. Data are expressed as the mean ± standard deviation (SD) or median, *P* < 0.05 was considered statistically significant. Graphs were plotted using the GraphPad Prism 9 software (GraphPad Software Inc., San Diego, CA, USA).

## 3 Results

### 3.1 Clinical features of included individuals

**Figure 1:**
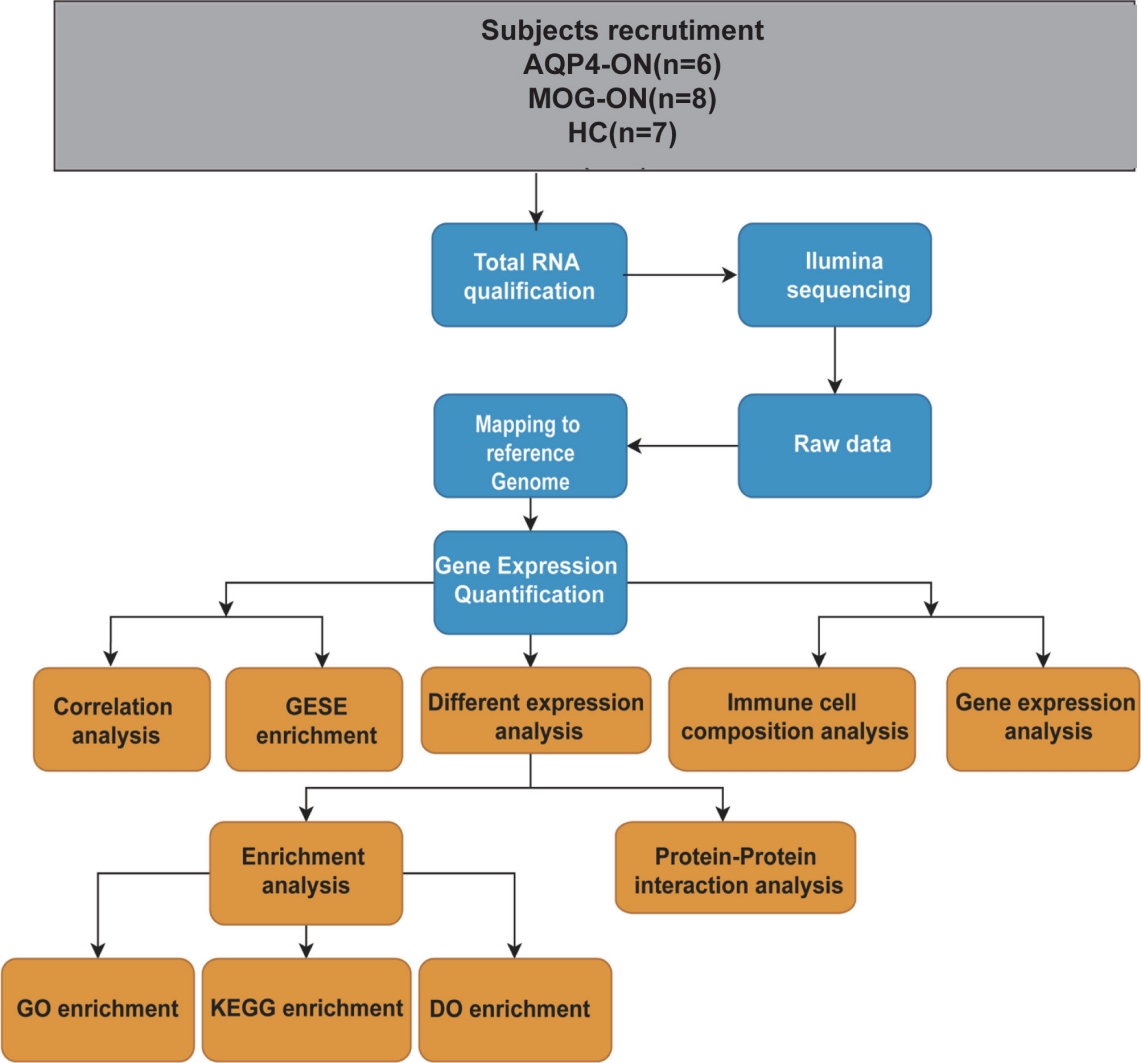
A workflow of this study. Age (years): age at the time of blood sample collection. Antibody titers: anti-AQP4 antibody titers detected by ELISA and the anti-MOG antibody titers detected by a CBA before blood samples were collected.

**Figure 2:**
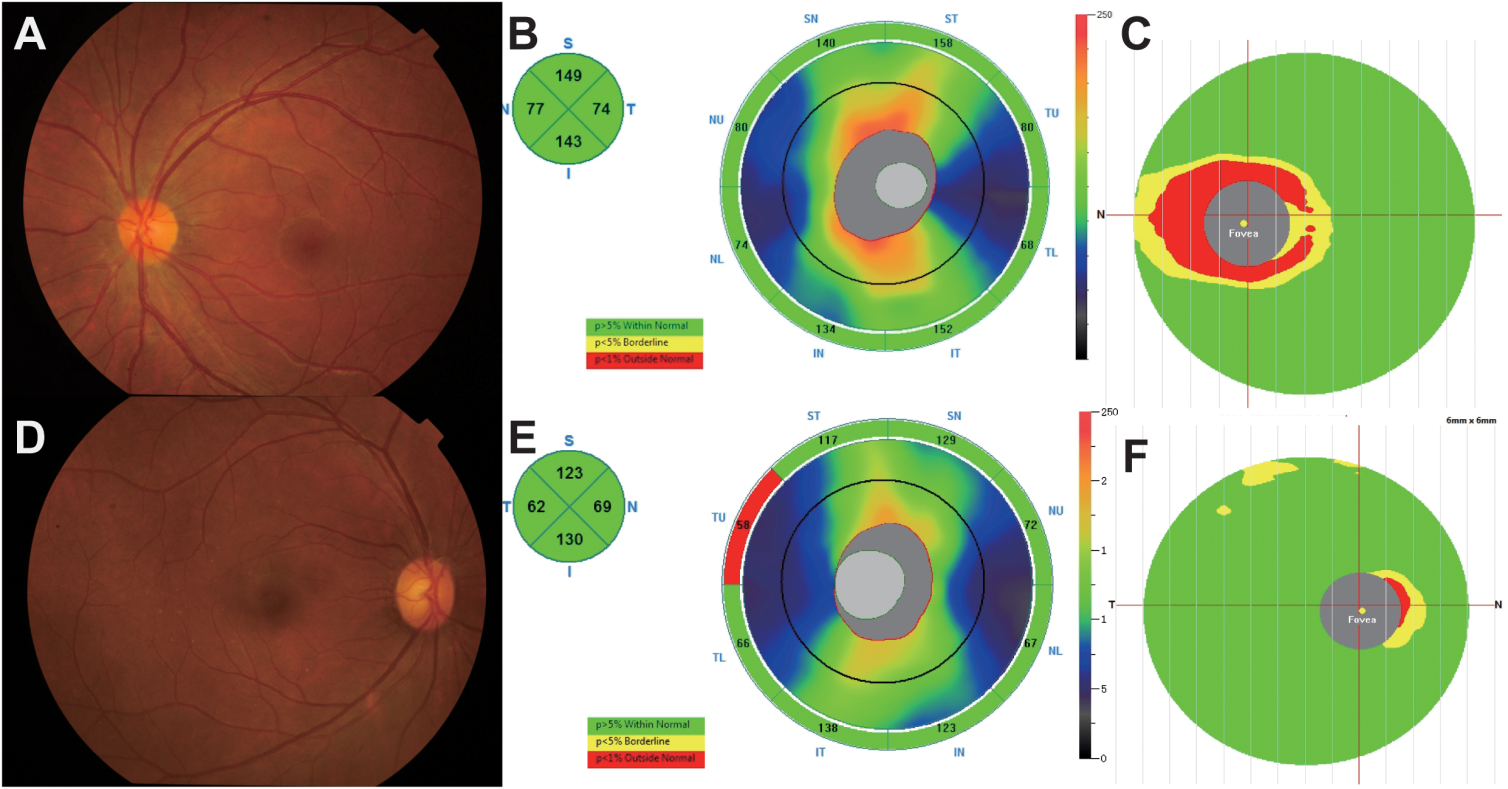
**(A–C)** A 25-year-old female complained of blurred vision in her left eye for 1 day. The BCVA (LogMAR) on presentation was 0.1 OD (Oculus Dexter) and 2 OS (Oculus Sinister). Fundus examination **(A)** showed a mildly edematous optic disc on OS, and optical coherence tomography (OCT) showed slight thickening of the retinal nerve fiber layer (RNFL) and thinning of the ganglion cell complex (GCC) at 1 month **(B, C)**. The anti-AQP4 antibody titer in this patient was 79.9 u/ml. **(D–F)** A 39-year-old female presented to our hospital with blurred vision in both eyes for 1 month, and the BCVA (logMAR) was 0 OU (oculus uterque). According to fundus photographs **(D)**, the optic disc of the right eye was almost normal, and the thicknesses of the RNFL and GCC were within the normal range **(E, F)**. The titer of the anti-MOG antibody in this patient was 1:10.

A flow chart of this study was shown in **Figure 1**. The clinical manifestations in 6 AQP4-ON patients, 8 MOG-ON patients and 7 HC subjects were shown in **Table 1**. The mean age was 37.83 ± 20.48 years for the AQP4-ON group, 38.25 ± 17.21 years for the MOG-ON group, and 41.42 ± 3.99 years for HC group. At the beginning of the disease, the BCVA (LogMAR) of the AQP4-ON group (2.13 ± 0.97) was slightly worse than which of the MOG-ON group (1.20 ± 0.71). The mean titer of anti-AQP4-IgG was 50.98 ± 29.35, and which of anti-MOG-IgG was 1:46.50 ± 44.92. In patients with AQP4-ON or MOG-ON, the thickness of the ganglion cell complex (GCC) and retinal nerve fiber layer (RNFL) became thinner as different degrees (**Figure 2**). In addition, one AQP4-ON patient and one MOG-ON patient were positive for antinuclear antibodies (ANA), and one MOG-ON patient had hypothyroidism.

**Table 1:** Demographic data of AQP4-ON and MOG-ON patients and healthy controls (HCs).

	AQP4-ON	MOG-ON	HC	*P* value
Number	6	8	7	–
Sex (female/male)	6/0	5/3	5/2	0.36
Age(years)	37.83±20.48	38.25±17.21	41.42±3.99	0.90
IOP(mmHg)	15.75±5.74	16.15±3.67	NA	0.88
BCVA at diagnosis	2.13±0.97	1.20±0.71	NA	0.06
BCVA at the last follow-up	0.78±1.34	0.27±0.29	NA	0.31
AQP4 antibody titers	50.98±29.35	–	NA	–
MOG antibody titers	–	46.50±44.92	NA	–

Age: years at diagnosis; IOP, Intra-Ocular Pressure; BCVA: Snellen visual acuity was converted to logarithm of the minimum angle of resolution (logMAR). Counting fingers vision was converted to a value of 2.0 logMAR, hand motion vision was converted to a value of 2.5 logMAR, light perception vision was converted to a value of 3.0 logMAR and no light perception vision was converted to a value of 3.5 logMAR. NA, Not applicable.

### 3.2 Overview of RNA-Seq data and differential gene expression profiles

**Figure 3:**
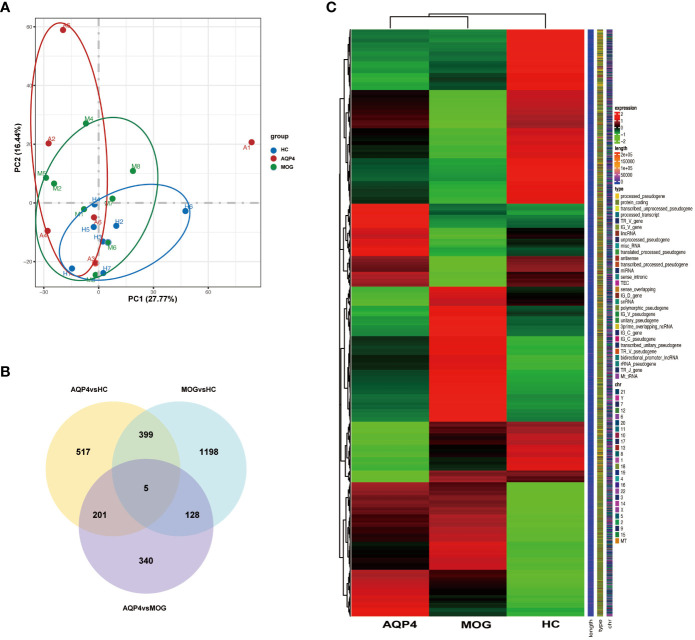
**(A)** Principal component analysis (PCA). The green, red and blue dots represent the HC, AQP4-ON, and MOG-ON groups, respectively. **(B)** Co-expression Venn diagram. **(C)** Cluster map of FPKM value of DEGs. The abscissa is the group name, and the ordinate is the gene name. The redder the color, the higher the expression level, and the greener the color, the lower the expression level.

**Figure 4:**
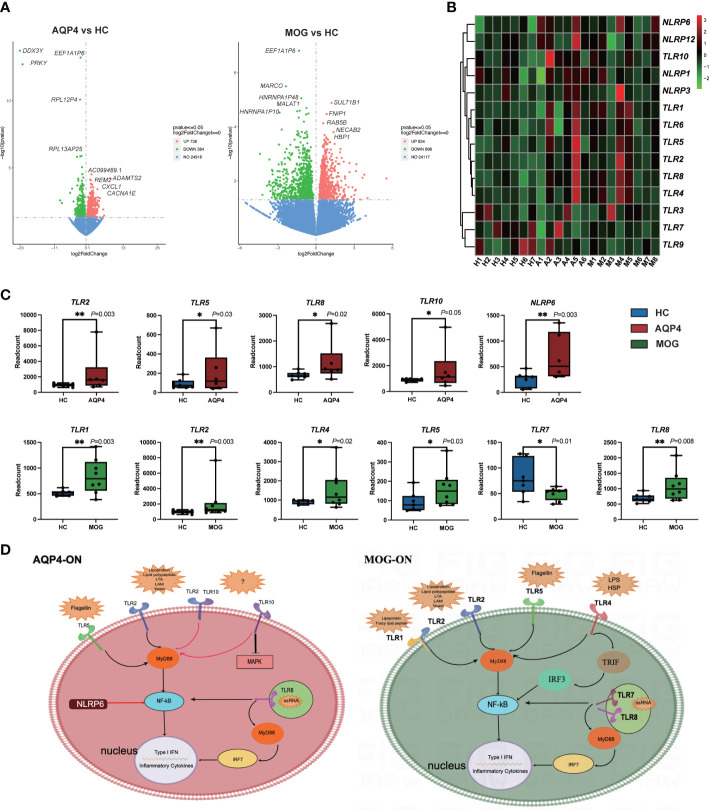
**(A)** Volcano plots showing the DEGs in the AQP4-ON group and MOG-ON group. The left panel is the volcanic map of AQP4-ON group vs HC group, and the right is the volcanic map of the MOG-ON group vs HC group. Red and green dots represent up-regulated and down-regulated genes, respectively. **(B)** is the cluster map of FPKM value of the TLR genes. **(C)** The box plot of the TLRs and NLRs of the AQP4-ON and MOG-ON groups according to the result of the statistical analysis of DEGs. The symbol * indicates that the p-value is less than 0.05, and the symbol ** indicates that the p-value is less than 0.01. **(D)** was according to the DEG analysis, GO term enrichment analysis and KEGG pathway enrichment analysis, AQP4-ON and MOG-ON activate the NF-κB pathway through the promotion of the expression of different TLRs and NLRs. The mechanism plot was drawn using the online tool Figdraw (www.figdraw.com). Red arrows indicate inhibition and black arrows indicate promotion.

The gene expression matrices of AQP4-ON and MOG-ON patients and HC subjects were standardized, and the principal component analysis (PCA) plot was drawn (**Figure 3A**). The PCA results revealed (PC1 = 30.95%) that there was no significant difference among the three groups. Venn diagram showed that there were five co-expressed genes among the three groups, 517 unique genes in the AQP4-ON group and 1,198 unique genes in the MOG-ON group (**Figure 3B**). Heatmap analysis showed that there were significant differences in gene expression among the three groups (**Figure 3C**). For all DEGs, see **Supplementary Document 1**. Further analysis of the DEGs among the three groups was conducted (**Figure 4**). In the AQP4-ON group, the expressions of *AC099489.1*, *ADAMTS2*, *REM2*, *CXCL1* and *CACNA1E* were significantly increased, while the expressions of *DDX3Y*, *EEF1A1P6*, *PRKY*, *RPL2P4* and *RPL13AP25* were significantly decreased, compared to the HC group. In MOG-ON group, the expressions of *SULT1B1*, *FNIP1*, *RAB5B*, *NECAB2*, *HBP1* were significantly increased, and the expressions of *EEF1A1P6*, *MARCO*, *HNRNP1AP48*, *MALAT1* and *HNRNP1AP10* were significantly decreased, compared to the HC group. Furthermore, due to the important roles in innate immunity and adaptive immunity, the expression of toll-like receptors (TLRs) was compared among the three groups. Compared to the HC group, *TLR2*, *TLR5*, *TLR8* and *TLR10* were up-regulated in the AQP4-ON group, and *TLR1*, *TLR2*, *TLR4*, *TLR5* and *TLR8* were up-regulated in the MOG-ON group. In addition, it was also found that the expression of *NLRP6* in AQP4-ON was increased and the expression of *TLR7* in MOG-ON was decreased. The differential expression of TLRs and NODs might indicate the different immunological mechanism involved in the pathogenesis of AQP4-ON and MOG-ON (**Figure 4C**).

### 3.3 Biological significance of DEGs

**Figure 5:**
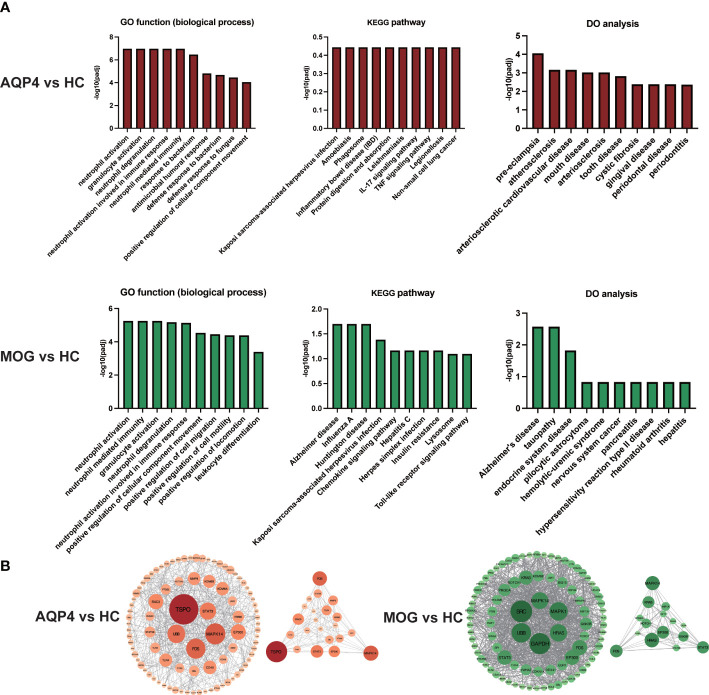
**(A)** GO functional enrichment analysis, KEGG pathway enrichment analysis and DO enrichment analysis of DEGs in the AQP4-ON group and MOG-ON group. **(B)** PPI analysis of DEGs in the AQP4-ON group and MOG-ON group.

For the biological functional analysis of DEGs, GO term enrichment analysis, KEGG pathway enrichment analysis and DO enrichment analysis were used to identify enriched GO functional categories (Biological Process (BP)), KEGG pathways and human diseases for DEGs. According to the GO term enrichment analysis of DEGs associated with the 10 most significant GO terms (**Figure 5**), the DEGs in AQP4-ON were likely associated with damage-associated molecular pattern (DAMP)-mediated inflammation, including neutrophil degranulation, neutrophil activation involved in immune response and neutrophil mediated immunity, and the DEGs in MOG-ON were likely associated with pathogen-associated molecular pattern (PAMP)-mediated inflammation, including activation of innate immune response, regulation of innate immune response and other biological functions. According to KEGG pathway enrichment analysis of the DEGs in AQP4-ON, they were mainly related to acquired immunity, including IL-17 signaling pathway, TNF signaling pathway and Th1 and Th2 cell differentiation. The corresponding KEGG enrichment analysis of the DEGs in MOG-ON revealed that MOG-ON signaling pathways mainly involved Influenza A, Kaposi sarcoma-associated herpesvirus infection, Herpes simplex infection and Toll-like receptor signaling pathway. According to DO enrichment analysis, the AQP4-ON group was mainly associated with atherosclerosis, arteriosclerotic cardiovascular disease and arteriosclerosis, while the MOG-ON group was associated with Alzheimer’s disease, hypersensitivity reaction type II disease and rheumatoid arthritis. The prediction of the interaction between proteins performed by PPI analysis in the AQP4-ON group using the STRING database revealed that *TSPO* and *MAPK14* were the core genes (**Figure 5B**). Translocator protein (TSPO) has been found to be up-regulated in inflammation diseases of central nervous system, and according our results, TSPO may be an important target for AQP4-ON (16). *MAPK14* is an important player in a variety of nervous system diseases, and contributes to the control of cerebrovascular and blood-brain barrier. The disruption of the blood-brain barrier in AQP4-ON may be related to the activation of *MAPK14* (17). Meanwhile the PPI analysis in the MOG-ON group identified *UBB* and *MAPK14* as the most significant targets. *UBB* is an important gene encoding ubiquitin, and ubiquitination plays an important role in biological functions such as antigen presentation, immune response and inflammation, and according our results, UBB may be an important target for MOG-ON (18).

### 3.4 Cell type analysis based on gene expression signature

**Figure 6:**
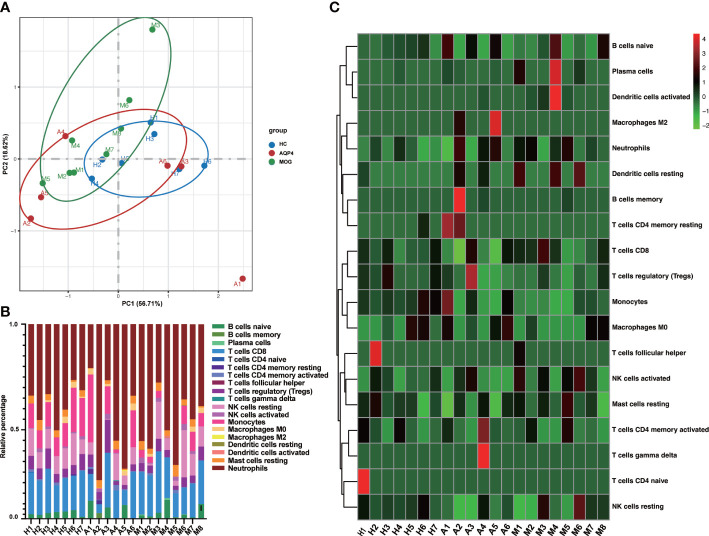
**(A)** PCA based on CIBERSORTx results. **(B)** The cumulative histogram of the proportion of 22 immune cell subtypes in each sample, and the total proportion of the 22 immune cell subtypes is equal to 100%. **(C)** The specific heat map analysis of the 22 immune cell subtypes in each sample.

The proportion of immune cells in the three groups showed individual differences (**Supplementary Table 1**). The 22 types of immune cells analyzed by CIBERSORTx showed no Macrophages M1, mast cells activated and eosinophils infiltration in any subject, and T cells CD4 naïve was only found in one healthy control. Neutrophils were the largest subset of cells in the subjects (22.6%~80.1%). B cells memory infiltration was found only in A2, A4 and M5, T cells CD4 memory resting was found only in A1, A2 and a healthy control, T cells gamma delta was found only in A4, and Macrophages M2 was found only in A2 and A5. Finally, we found that Dendritic cells resting and Dendritic cells activated existed almost in ON patients. Then, the PCA based on the results of the CIBERSORTx analysis revealed that except for the sample from patient A1, the other samples showed obvious intra group clustering and inter group differences (**Figure 6A**). (**Figure 6B**, **C**) showed that there are differences in the proportion of immune cells among the three groups

### 3.5 Correlation between immune cell infiltration and clinical outcomes

**Figure 7:**
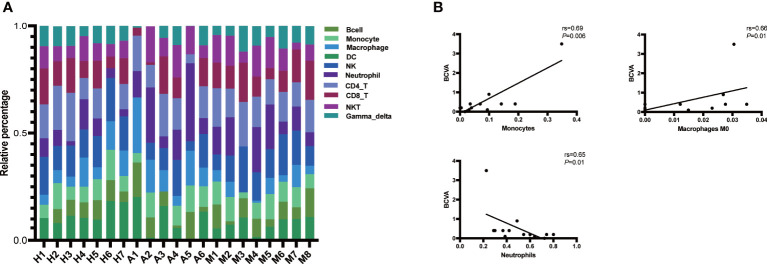
**(A)** Immune cell abundance analysis was performed on all patients and subjects using the ImmuCellAI software. Histogram of the proportions of the 24 immune cells in the AQP4-ON group, MOG-ON group and HC group. X-axis: ID of each subject; Y-axis: percentage of immune cell types. Gamma_delta: γδ T cell, NKT, natural killer T cell; CD8_T, CD8 + T cell; CD4_T, CD4 + T cell; NK, natural killer T cell; DC, dendritic cell. **(B)** Spearman correlation analysis of the relationship be-tween immune cells and BCVA (LogMAR) at diagnosis. rs is the Spearman correlation coefficient, negative number represents negative correlation and positive number represents positive correlation. *P* < 0.05 indicates statistical significance.

The results of the ImmuCellAI analysis (**Figure 7A**) for AQP4-ON, MOG-ON and HC group revealed that although there were differences between the two methods in the classification of immune cells, the proportion of infiltrated immune cell types was roughly similar to that of CIBERSORTx. Thus, we analyzed the correlation between the proportion of infiltrated immune cell types and the clinical results of patients. A total of 14 patients were included in this study, and the therapeutic regimen as well as the BCVA(LogMAR) at the last follow up were shown in **Table 2**. The patient A4 refused to be treated, and the BCVA (LogMAR) of the Oculus Dexter (OD) recovered to normal levels in about 2 months after the onset, and no recurrence has occurred to date. The data in **Figure 5B** revealed that there was almost no B cell infiltration in the peripheral blood, which might explain the good prognosis of this patient. The AQP4-IgG titer of patient A2 was only 10.37 u/ml; however, despite the IVMP and oral prednisolone treatment, her vision was still very poor. It showed that the proportion of neutrophil infiltration accounted for the largest proportion of all subjects, which might indicate that her severe inflammatory rection was the cause of irreversible damage of her vision. The Spearman correlation analysis of the relationship between the 22 kinds of immune cells and patients’ BCVA (LogMAR) at diagnosis (**Figure 7**) showed that the infiltration ratios of monocytes (rs=0.69, *P*=0.006) and M0 macrophages (rs=0.66, *P*=0.01) were positively correlated with the BCVA (LogMAR). Moreover, the infiltration ratio of neutrophils was negatively correlated with the BCVA (LogMAR) (rs=0.65, *P*=0.01). No significant correlation was found among the other 18 immune cell subtypes (data not shown).

**Table 2:** Treatment and prognosis of patients.

Patient ID	Age(years)	Sex	Antibody titer	Affected eyes	Therapeutic methods	BCVA at diagnosis	Last follow-up BCVA
A1	71	Female	AQP4-IgG:>80u/ml	Right	IVMP and oral prednisolone	3.5	3.5
A2	45	Female	AQP4-IgG:10.37u/ml	Left	Oral prednisolone	1.7	2
A3	11	Female	AQP4-IgG:67.90u/ml	Right	IVMP and oral prednisolone	2	0.4
A4	43	Female	AQP4-IgG:40.83u/ml	Right	None	0.6	0
A5	32	Female	AQP4-IgG:>80u/ml	Right	IVMP and oral prednisolone	2.5	0.2
A6	25	Female	AQP4-IgG:26.8u/ml	Left	IVMP and oral prednisolone	2.5	0.4
M1	18	Male	MOG-IgG:1:10	Left	IVMP and oral prednisolone	0.4	0.2
M2	32	Female	MOG-IgG:1:10	Left	IVMP and oral prednisolone	0.5	0.1
M3	57	Female	MOG-IgG:1:10	Left	IVMP and oral prednisolone	1.1	0.4
M4	24	Female	MOG-IgG:1:100	Left	IVMP and oral prednisolone	1.1	0.2
M5	39	Female	MOG-IgG:1:10	Right	IVMP and oral prednisolone	2	0
M6	70	Female	MOG-IgG:1:100	Left	IVMP and oral prednisolone	2	0.1
M7	35	Male	MOG-IgG:1:32	Right	IVMP and oral prednisolone	2	0.9
M8	31	Male	MOG-IgG:1:100	Right	IVMP and oral prednisolone	0.5	0.2

Age: years at diagnosis; IVMP, intravenous pulses of methylprednisolone. BCVA: Snellen visual acuity was converted to logarithm of the minimum angle of resolution (logMAR). Counting fingers vision was converted to a value of 2.0 logMAR, hand motion vision was converted to a value of 2.5 logMAR, light perception vision was converted to a value of 3.0 logMAR and no light perception vision was converted to a value of 3.5 logMAR.

## 4 Discussion

Currently, most researchers in the field believe that although MOG-ON is similar to AQP4-ON in clinical manifestations, unlike AQP4-ON, MOG-ON is not an immune subtype of neuromyelitis optica spectrum disorder (NMOSD), but a subtype of MOG antibody-related diseases (MOGAD) (19). Moreover, magnetic resonance imaging (MRI) reveals obvious differences between the cortical grey/juxtacortical white matter lesions on the brain of patients with MOGAD and those of patients with AQP4-IgG seropositive NMOSD (20). This finding indicates that the immunological mechanism of AQP4-ON and MOG-ON may be different. In this study, the RNA-seq analysis of whole blood from AQP4-ON and MOG-ON patients and HC subjects revealed that there were significant differences in gene expression and cell function among the three groups. In particular, the results suggested that the AQP4-ON patients likely presented PAMP-induced inflammation, and MOG-ON patients likely presented DAMP-induced inflammation. In addition, cell type analysis based on gene expression signature showed that the proportion of immune cells was similar in the HC group, but was significantly different between the AQP4-ON group and MOG-ON group.

DAMPs are endogenous molecules released from the cell, which serve as potent activators of the immune system (21). Cellular stressors, including physical (trauma, radiation), chemical (toxins, osmolarity), metabolic (ischemia/reperfusion), and infectious (viruses, bacteria, protozoa) factors, can trigger DAMP release (21). DAMPs are systemically and locally up-regulated in autoimmune diseases, such as rheumatoid arthritis. PAMPs are derived from microorganisms and recognized by pattern recognition receptor (PRR)-bearing cells of the innate immune system (22). However, the roles of DAMPs and PAMPs in AQP4-ON and MOG-ON have never been reported. Biological function analysis of DEGs revealed that DEGs in AQP4-ON patients were mainly involved in the regulation of inflammatory cells, such as regulation of granular chemotaxis, positive regulation of leucocyte chemotaxis, regulation of leucocyte chemotaxis. and these over-represented terms are closely related to DAMP-induced adaptive immunity. The analysis also revealed that DEGs in MOG-ON patients were mainly involved in the activation of inflammatory cells, such as neutrophil activation, granulocyte activation, neutrophil activation involved in immune responses, and the reaction to bacteria and viruses, such as response to bacteria, defense response to bacterium, response to LPS, and these enriched terms fully indicate that MOG-ON is closely related to PAMP-induced innate immunity. Currently, PAMPs and DAMPs are known to bind to the receptors of TLRs to modulate their activation and promote the synthesis of cytokines. TLRs are type I transmembrane pattern recognition receptors which can recognize molecules present on the surface of pathogen cells (23). Ten types of TLRs have been identified in human, with different ligands. TLRs are widely distributed on the surface of macrophages, dendritic cells and epithelial cells. It has been found that the percentage of peripheral CD4^+^ T cells expressing TLR2, TLR4 and TLR9 in NMOSD samples was significantly higher than that in healthy subjects (24). According to our DEGs analysis, the expression of TLRs was clearly different between the AQP4-ON and MOG-ON groups. Specifically, *TLR2*, *TLR5*, *TLR8* and *TLR10* were significantly up-regulated in the AQP4-ON group, while *TLR1*, *TLR2*, *TLR4*, *TLR5* and *TLR8* were significantly up-regulated in the MOG-ON group. Both the *TLR2* and *TLR5* are only expressed on the cell surface of myeloid cells, such as monocytes and macrophages, while *TLR7* is found in intracellular vesicles, where it is involved in identifying the nucleic acid components of microorganisms, and all of them can activate NF-κB. TLR10 is the most recently identified human TLR, and its function and ligand are still unclear. TLR10 can inhibit other TLRs by competing with dimers of TLR1, TLR2 and TLR6, which can induce PI3K/AKT signaling, but other studies have reported that TLR10 may stimulate and amplify the activity of TLR2 when it forms an isomeric dimer with TLR2 (25). However, the detailed functions of TLR10 in AQP4-ON need to be further investigated in animal models. TLR4 is the main receptor of LPS, an important PAMP. The binding of LPS to TLR4 activates signal transduction pathways in two ways, namely through toll-interleukin-1 receptor domain containing adaptor protein (TIRAP) and MyD88. TLR2/TLR4 have emerged as targets for treating a wide array of autoimmune disorders. Liu et al. reported that blocking TLR2/TLR4 in the experimental autoimmune encephalomyelitis (EAE) model of multiple sclerosis prevents the production of proinflammatory factors, which is consistent with our clinical data (26). In addition to TLRs, Nod-like receptors (NLRs) are also important PRRs for identifying PAMP and DAMP. Our results showed that NLRP6 was significantly up-regulated in AQP4-ON, which is different from other pro-inflammatory mechanisms initiated by NLRs (27).

At present, it has been confirmed that T, NK and B cells play important roles in the pathogenesis of AQP4-ON and MOG-ON. The B cell-mediated humoral immune response by autoreactive IgG antibodies against AQP4 or MOG is considered to be the driver of NMOSD. Recently, other immune cells, such as T cells, have also attracted the attention of researchers. However, their detailed functions remain unclear. A previous study by Zhou et al. analyzed non-coding RNAs in peripheral blood mononuclear cells (PBMCs) from NMOSD patients using the online web tool xCell, and found that the immune scores of CD8+ T cells, M1 macrophages and plasma cells in the NMOSD group were increased, while that of M2 macrophages was decreased (28). The traditional method uses immunohistochemistry to infer immune subsets of cells to evaluate immune cell tissue invasion, but such a method is often useful for solid tumors (29). At present, flow cytometry is widely used for counting mixed cells, which is an alternative method for quantitating immune infiltration that can simultaneously measure multiple parameters (30). However, this method requires processing samples in a timely and precise manner, which may lead to loss of some cell types and distortion of GEPs. Unlike flow cytometry, which relies on cell surface marker antibodies as the basis for cell classification, our study applied CIBERSORTx to quantify cell fractions from GEPs using a deconvolution algorithm based on peripheral blood RNA-seq data, and then accurately estimated the proportion of infiltrated immune cells. CIBERSORTx immune cell infiltration results were verified by fluorescent activated cell classification, and have been widely used in a variety of tumors and immune diseases. Our analysis of the correlation between the proportion of 22 types of immune cells and BCVA (LogMAR) of patients at the last follow-up showed that the ratio of monocytes or M0 macrophages was negatively correlated with BCVA (LogMAR), while the ratio neutrophils was positively correlated with BCVA (LogMAR). In other words, the infiltration ratio of monocytes or M0 macrophages was negatively correlated with the prognosis of the visual acuity of patients, and the infiltration ratio of neutrophils was positively correlated with the prognosis of the visual acuity of patients. Therefore, our study revealed that monocytes, M0 macrophages and neutrophils might serve as indicators of the visual prognosis of patients.

Over all, this study revealed different immunological mechanisms between AQP4-ON and MOG-ON patients based on transcriptomics analysis of patients’ whole blood, and found the proportion of immune cell infiltration was closely related to patients’ vision. However, there are still some limitations to this study. First, the sample size is still small, which may lead to the deviation of the results, and it needs to expand the sample size in the further study. Then, future research is warranted to investigate the detailed pathological mechanism responsible for AQP4-ON and MOG-ON using *in vitro* and *in vivo* models based on transcriptomics data; however, this is beyond the scope of the current study.

## 5 Conclusion

In conclusion, our study shows that AQP4-ON and MOG-ON differ in the immune mechanism of activation of different TLR-related pathways as well as the infiltration of different immune cells, which not only provides novel insights into the pathogenic mechanism of AQP4-ON and MOG-ON, but also clues for the development of new therapeutic approaches for these diseases.

## Data availability statement

The datasets presented in this study can be found in online repositories. The names of the repository/repositories and accession number(s) can be found below: GSE226808 (GEO).

## Ethics statement

The studies involving human participants were reviewed and approved by The ethics committee of the Affiliated Wuxi Clinical College of Nantong University. Written informed consent to participate in this study was provided by the participants’ legal guardian/next of kin. Written informed consent was obtained from the [individual(s) AND/OR minor(s)’ legal guardian/next of kin] for the publication of any potentially identifiable images or data included in this article.

## Author contributions

Study conception and design: WZ, and KW. Collection of samples: XC, LC, YP, PC, YL, and SL. Data analysis: XC and LC. Drafting of manuscript and figures: XC and LC. Critical revision of the manuscript: WZ and KW. Statistical analysis: YP and PC. Obtained funding: WZ. All authors contributed to the article and approved the submitted version.
