# Corrigendum: Different immunological mechanisms between AQP4 antibody-positive and MOG antibody-positive optic neuritis based on RNA sequencing analysis of whole blood

**DOI:** 10.3389/fimmu.2023.1201718

**Published:** 2023-08-15

**Authors:** Xuelian Chen, Libo Cheng, Ying Pan, Peng Chen, Yidan Luo, Shiyi Li, Wenjun Zou, Ke Wang

**Affiliations:** ^1^Department of Ophthalmology, Affiliated Wuxi Clinical College of Nantong University, Wuxi, Jiangsu, China; ^2^Department of Ophthalmology, Jiangnan University Medical Center (JUMC), Wuxi, Jiangsu, China; ^3^Department of Ophthalmology, Wuxi No.2 People’s Hospital, Wuxi, Jiangsu, China; ^4^Department of Ophthalmology, The Affiliated Wuxi No.2 People’s Hospital of Nanjing Medical University, Wuxi, Jiangsu, China; ^5^National Health Commission (NHC) Key Laboratory of Nuclear Medicine, Jiangsu Key Laboratory of Molecular Nuclear Medicine, Jiangsu Institute of Nuclear Medicine, Wuxi, Jiangsu, China; ^6^Department of Radiopharmaceuticals, School of Pharmacy, Nanjing Medical University, Nanjing, Jiangsu, China

**Keywords:** optic neuritis, aquaporin 4, myelin oligodendrocyte glycoprotein, RNA-Seq, toll-like receptors

In the published article, there were errors in **Figure 2** and its legend. In the published article, parts A –C of **Figure 2** for AQP4-ON patient should be consistent with the course of MOG-ON patient for one month. The corrected **Figure 2** and its legend appear below.

**Figure 2:**
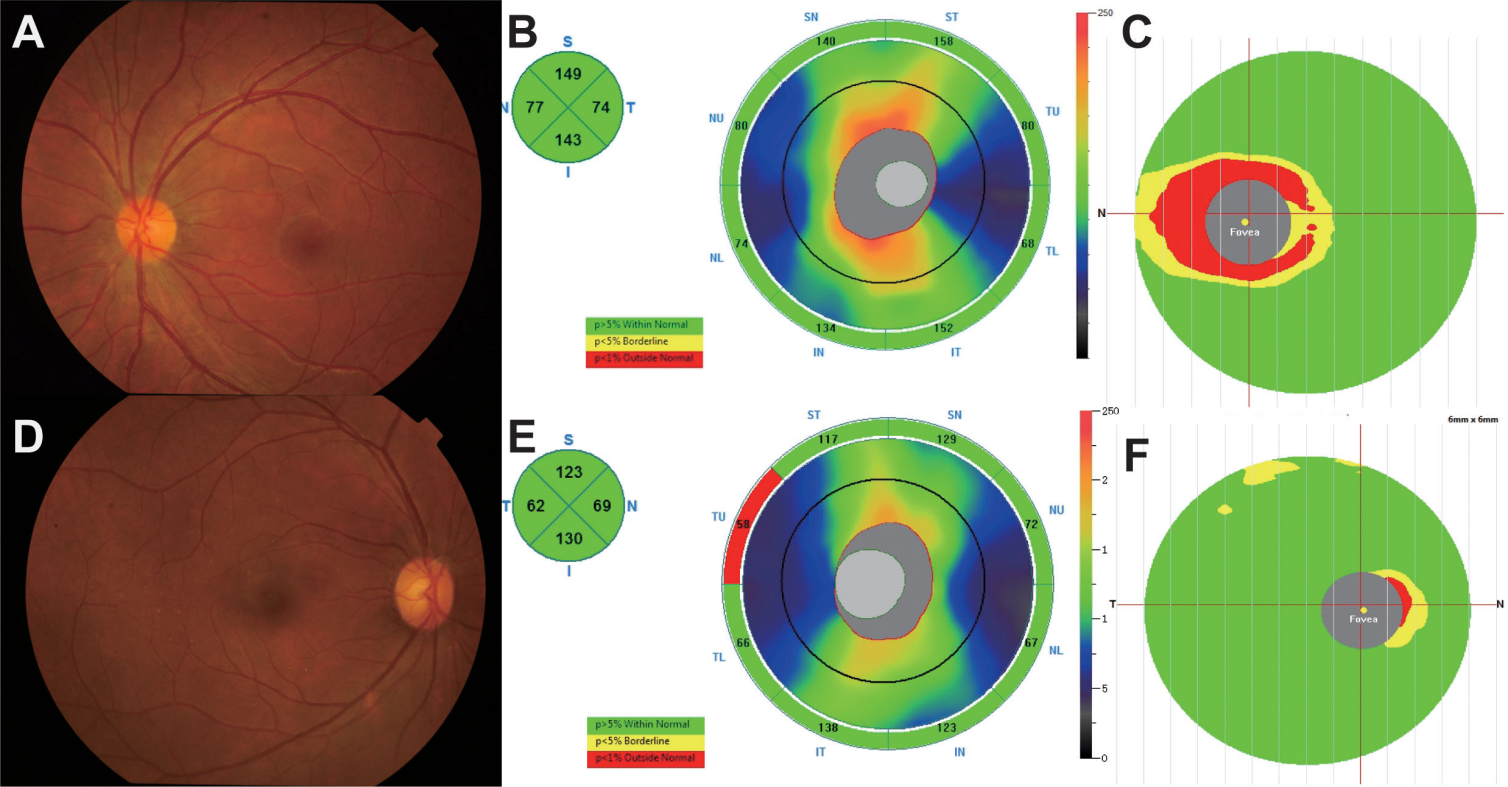
**(A–C)** A 25-year-old female complained of blurred vision in her left eye for 1 day. The BCVA (LogMAR) on presentation was 0.1 OD (Oculus Dexter) and 2 OS (Oculus Sinister). Fundus examination **(A)** showed a mildly edematous optic disc on OS, and optical coherence tomography (OCT) showed slight thickening of the retinal nerve fiber layer (RNFL) and thinning of the ganglion cell complex (GCC) at 1 month **(B, C)**. The anti-AQP4 antibody titer in this patient was 79.9 u/ml. **(D–F)** A 39-year-old female presented to our hospital with blurred vision in both eyes for 1 month, and the BCVA (logMAR) was 0 OU (oculus uterque). According to fundus photographs **(D)**, the optic disc of the right eye was almost normal, and the thicknesses of the RNFL and GCC were within the normal range **(E, F)**. The titer of the anti-MOG antibody in this patient was 1:10.

In the published article, there was an error in **Table 2**. In the published article, the unit of AQP4-IgG titer should be u/ml. The initial letter of the content of M8 patients should be capitalized, and the antibody titer result should be 1:100 instead of 1; 100. The corrected **Table 2** and its captions are listed below.

**Table 2:** Treatment and prognosis of patients.

Patient ID	Age(years)	Sex	Antibody titer	Affected eyes	Therapeutic methods	BCVA at diagnosis	Last follow-up BCVA
A1	71	Female	AQP4-IgG:>80u/ml	Right	IVMP and oral prednisolone	3.5	3.5
A2	45	Female	AQP4-IgG:10.37u/ml	Left	Oral prednisolone	1.7	2
A3	11	Female	AQP4-IgG:67.90u/ml	Right	IVMP and oral prednisolone	2	0.4
A4	43	Female	AQP4-IgG:40.83u/ml	Right	None	0.6	0
A5	32	Female	AQP4-IgG:>80u/ml	Right	IVMP and oral prednisolone	2.5	0.2
A6	25	Female	AQP4-IgG:26.8u/ml	Left	IVMP and oral prednisolone	2.5	0.4
M1	18	Male	MOG-IgG:1:10	Left	IVMP and oral prednisolone	0.4	0.2
M2	32	Female	MOG-IgG:1:10	Left	IVMP and oral prednisolone	0.5	0.1
M3	57	Female	MOG-IgG:1:10	Left	IVMP and oral prednisolone	1.1	0.4
M4	24	Female	MOG-IgG:1:100	Left	IVMP and oral prednisolone	1.1	0.2
M5	39	Female	MOG-IgG:1:10	Right	IVMP and oral prednisolone	2	0
M6	70	Female	MOG-IgG:1:100	Left	IVMP and oral prednisolone	2	0.1
M7	35	Male	MOG-IgG:1:32	Right	IVMP and oral prednisolone	2	0.9
M8	31	Male	MOG-IgG:1:100	Right	IVMP and oral prednisolone	0.5	0.2

In the published article, there was an error in the description of **Figure 3B**.

A correction was made to the **Results**, *Overview of RNA-Seq data and differential gene expression profiles* (Paragraph 3.2). This sentence was previously stated as follows:

“[Venn diagram showed that there were 10 co-expressed genes among the three groups, 924 unique genes in the AQP4-ON group and 502 unique genes in the MOG-ON group (**Figure 3B**).]”

**Figure 3:**
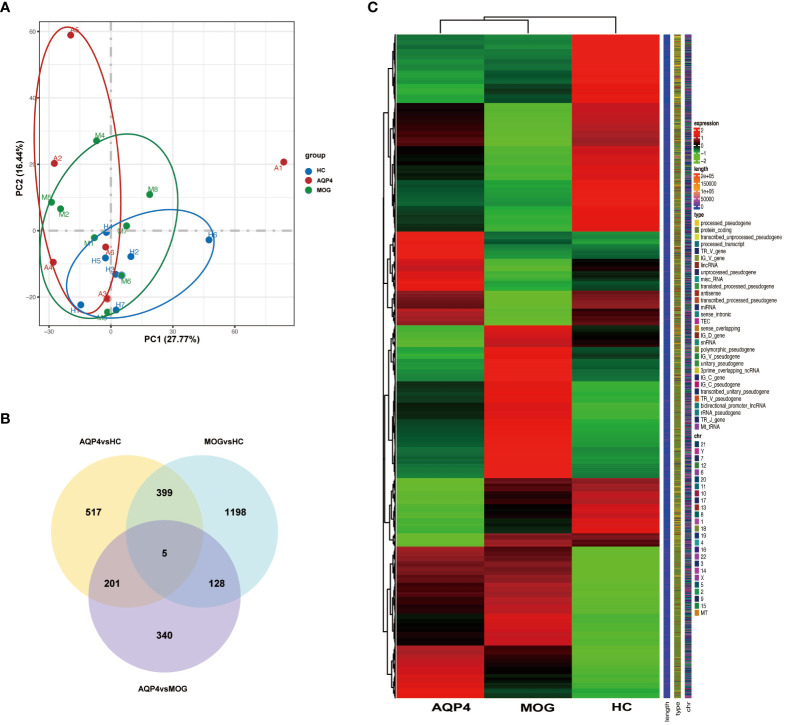
**(A)** Principal component analysis (PCA). The green, red and blue dots represent the HC, AQP4-ON, and MOG-ON groups, respectively. **(B)** Co-expression Venn diagram. **(C)** Cluster map of FPKM value of DEGs. The abscissa is the group name, and the ordinate is the gene name. The redder the color, the higher the expression level, and the greener the color, the lower the expression level.

The corrected sentence is as follows:

“[Venn diagram showed that there were five co-expressed genes among the three groups, 517 unique genes in the AQP4-ON group and 1,198 unique genes in the MOG-ON group (**Figure 3B****)**.]”

A further correction has been made in the **Discussion** section, [Paragraph 4]. This sentence was previously stated as follows:

“[Currently, most researchers in the field believe that although MOG-ON is similar to AQP4-ON in clinical manifestations, unlike AQP4-ON, MOG-ON is not an immune subtype of neuromyelitis optical spectrum disorder (NMOSD), but a subtype of MOG antibody-related diseases (MOGAD).]”

The corrected sentence is as follows:

“[Currently, most researchers in the field believe that although MOG-ON is similar to AQP4-ON in clinical manifestations, unlike AQP4-ON, MOG-ON is not an immune subtype of neuromyelitis optica spectrum disorder (NMOSD), but a subtype of MOG antibody-related diseases (MOGAD).]”

In the published article, there was an error. [AQP4-IgG titer unit should be u/ml].

A correction was made to **[Materials and methods** and **Results]**, *[Subjects and samples* and *Correlation between immune cell infiltration and clinical outcomes***]**, [Paragraphs 2.1 and 3.5]. This sentence was previously stated as follows:

“[For high sensitivity and absolute specificity, the AQP4-IgG was measured using an enzyme-linked immunosorbent assay (ELISA) kit (RSR Ltd., Cardiff, UK) according to the previous study (12) and results 3.0 m/ml were considered as positive.

The AQP4-IgG titer of patient A2 was only 10.37 μ/ml; however, despite the IVMP and oral prednisolone treatment, her vision was still very poor.]”

The corrected sentence is as follows:

“[For high sensitivity and absolute specificity, the AQP4-IgG was measured using an enzyme-linked immunosorbent assay (ELISA) kit (RSR Ltd., Cardiff, UK) according to the previous study (12) and results 3.0 u/mL were considered as positive.

The AQP4-IgG titer of patient A2 was only 10.37 u/ml; however, despite the IVMP and oral prednisolone treatment, her vision was still very poor.]”

In the published article there was an error in the **Ethics** section. Due to potentially identifiable data included in the article, the following sentence has been included - “Written informed consent was obtained from the [individual(s) AND/OR minor(s)’ legal guardian/next of kin] for the publication of any potentially identifiable images or data included in this article.”

The new Ethics statement appears below.

## Ethics statement

The studies involving human participants were reviewed and approved by The ethics committee of the Affiliated Wuxi Clinical College of Nantong University. Written informed consent to participate in this study was provided by the participants’ legal guardian/next of kin. Written informed consent was obtained from the [individual(s) AND/OR minor(s)’ legal guardian/next of kin] for the publication of any potentially identifiable images or data included in this article.

The authors apologize for these errors and state that these do not change the scientific conclusions of the article in any way. The original manuscript has been updated accordingly.

