# Supplementary material for: Different immunological mechanisms between AQP4 antibody-positive and MOG antibody-positive optic neuritis based on RNA sequencing analysis of whole blood
Source: Front Immunol. 2023 Mar 9;14:1095966. doi: 10.3389/fimmu.2023.1095966 (PMC10036921; doi:10.3389/fimmu.2023.1095966)
Supplement: Supplementary file 2 [file Table_2.docx]

**Supplement Table 2.** Proportion of immune cells in each sample

| **Mixture** | **H1** | **H2** | **H3** | **H4** | **H5** | **H6** | **H7** | **A1** | **A2** | **A3** | **A4** | **A5** | **A6** | **M1** | **M2** | **M3** | **M4** | **M5** | **M6** | **M7** | **M8** |
| --- | --- | --- | --- | --- | --- | --- | --- | --- | --- | --- | --- | --- | --- | --- | --- | --- | --- | --- | --- | --- | --- |
| B cells naive | 2.36% | 1.94% | 2.96% | 3.34% | 3.73% | 4.42% | 0.96% | 9.15% | 0.00% | 5.76% | 0.00% | 7.00% | 0.24% | 1.71% | 1.21% | 3.06% | 9.63% | 0.48% | 2.06% | 0.69% | 7.52% |
| B cells memory | 0.00% | 0.00% | 0.00% | 0.00% | 0.00% | 0.00% | 0.00% | 0.00% | 2.91% | 0.00% | 0.22% | 0.00% | 0.00% | 0.00% | 0.00% | 0.00% | 0.00% | 0.25% | 0.00% | 0.00% | 0.00% |
| Plasma cells | 0.00% | 0.00% | 0.00% | 0.06% | 0.00% | 0.00% | 0.00% | 0.10% | 0.05% | 0.00% | 0.00% | 0.05% | 0.00% | 0.42% | 0.00% | 0.00% | 0.97% | 0.00% | 0.17% | 0.00% | 0.00% |
| T cells CD8 | 21.44% | 17.68% | 22.77% | 13.62% | 17.06% | 17.75% | 23.64% | 13.78% | 4.49% | 28.17% | 14.36% | 7.55% | 23.80% | 21.79% | 21.00% | 31.57% | 20.96% | 12.31% | 13.76% | 17.30% | 22.46% |
| T cells CD4 naive | 0.19% | 0.00% | 0.00% | 0.00% | 0.00% | 0.00% | 0.00% | 0.00% | 0.00% | 0.00% | 0.00% | 0.00% | 0.00% | 0.00% | 0.00% | 0.00% | 0.00% | 0.00% | 0.00% | 0.00% | 0.00% |
| T cells CD4 memory resting | 0.00% | 0.00% | 0.00% | 0.00% | 0.00% | 0.69% | 0.00% | 2.53% | 2.01% | 0.00% | 0.00% | 0.00% | 0.00% | 0.00% | 0.00% | 0.00% | 0.00% | 0.00% | 0.00% | 0.00% | 0.00% |
| T cells CD4 memory activated | 1.47% | 0.70% | 0.00% | 1.57% | 0.50% | 0.00% | 0.47% | 1.06% | 0.34% | 0.00% | 2.74% | 0.58% | 0.47% | 0.53% | 1.25% | 0.00% | 0.00% | 1.74% | 0.27% | 0.42% | 0.00% |
| T cells follicular helper | 0.00% | 0.05% | 0.00% | 0.00% | 0.00% | 0.00% | 0.00% | 0.00% | 0.00% | 0.00% | 0.00% | 0.00% | 0.00% | 0.02% | 0.00% | 0.00% | 0.00% | 0.00% | 0.00% | 0.00% | 0.00% |
| T cells regulatory (Tregs) | 6.86% | 5.47% | 10.35% | 4.40% | 4.42% | 4.71% | 8.17% | 6.34% | 3.94% | 16.99% | 2.00% | 2.16% | 3.44% | 4.10% | 3.85% | 6.44% | 3.74% | 2.19% | 4.97% | 3.77% | 7.13% |
| T cells gamma delta | 0.00% | 0.00% | 0.00% | 0.00% | 0.00% | 0.00% | 0.00% | 0.00% | 0.00% | 0.00% | 0.41% | 0.00% | 0.00% | 0.00% | 0.00% | 0.00% | 0.00% | 0.00% | 0.00% | 0.00% | 0.00% |
| NK cells resting | 14.06% | 8.37% | 10.09% | 9.16% | 10.51% | 16.36% | 7.53% | 6.29% | 0.00% | 0.41% | 13.84% | 4.88% | 8.84% | 0.03% | 0.99% | 19.04% | 6.11% | 2.55% | 24.30% | 11.10% | 10.30% |
| NK cells activated | 0.21% | 1.91% | 1.28% | 0.00% | 0.87% | 0.70% | 1.62% | 0.00% | 1.52% | 2.60% | 0.32% | 0.00% | 0.00% | 2.80% | 0.50% | 2.03% | 0.00% | 2.12% | 3.20% | 1.49% | 0.00% |
| Monocytes | 12.59% | 9.27% | 12.50% | 9.68% | 12.04% | 22.70% | 19.63% | 34.86% | 0.42% | 14.16% | 1.71% | 0.00% | 18.92% | 3.93% | 3.08% | 3.50% | 0.00% | 0.00% | 9.38% | 9.92% | 6.91% |
| Macrophages M0 | 0.00% | 0.00% | 1.03% | 0.00% | 3.32% | 3.00% | 0.68% | 3.05% | 0.00% | 1.21% | 0.00% | 2.30% | 3.49% | 0.00% | 1.49% | 0.00% | 0.00% | 0.00% | 0.00% | 2.69% | 2.76% |
| Macrophages M1 | 0.00% | 0.00% | 0.00% | 0.00% | 0.00% | 0.00% | 0.00% | 0.00% | 0.00% | 0.00% | 0.00% | 0.00% | 0.00% | 0.00% | 0.00% | 0.00% | 0.00% | 0.00% | 0.00% | 0.00% | 0.00% |
| Macrophages M2 | 0.00% | 0.00% | 0.00% | 0.00% | 0.00% | 0.00% | 0.00% | 0.00% | 0.22% | 0.00% | 0.00% | 0.51% | 0.00% | 0.00% | 0.00% | 0.00% | 0.00% | 0.00% | 0.00% | 0.00% | 0.00% |
| Dendritic cells resting | 0.05% | 0.00% | 0.00% | 0.00% | 0.00% | 0.00% | 0.00% | 0.00% | 0.13% | 0.00% | 0.09% | 0.00% | 0.00% | 0.17% | 0.00% | 0.00% | 0.17% | 0.00% | 0.20% | 0.00% | 0.00% |
| Dendritic cells activated | 0.00% | 0.00% | 0.00% | 0.00% | 0.00% | 0.00% | 0.00% | 0.00% | 0.00% | 0.00% | 0.05% | 0.00% | 0.00% | 0.00% | 0.06% | 0.15% | 1.07% | 0.06% | 0.00% | 0.00% | 0.00% |
| Mast cells resting | 4.02% | 5.35% | 3.08% | 3.56% | 3.63% | 0.99% | 3.30% | 0.25% | 3.86% | 1.92% | 4.36% | 0.70% | 4.01% | 4.44% | 2.59% | 4.22% | 2.77% | 5.91% | 3.03% | 3.72% | 0.79% |
| Mast cells activated | 0.00% | 0.00% | 0.00% | 0.00% | 0.00% | 0.00% | 0.00% | 0.00% | 0.00% | 0.00% | 0.00% | 0.00% | 0.00% | 0.00% | 0.00% | 0.00% | 0.00% | 0.00% | 0.00% | 0.00% | 0.00% |
| Eosinophils | 0.00% | 0.00% | 0.00% | 0.00% | 0.00% | 0.00% | 0.00% | 0.00% | 0.00% | 0.00% | 0.00% | 0.00% | 0.00% | 0.00% | 0.00% | 0.00% | 0.00% | 0.00% | 0.00% | 0.00% | 0.00% |
| Neutrophils | 36.74% | 49.26% | 35.94% | 54.61% | 43.90% | 28.69% | 34.01% | 22.58% | 80.12% | 28.79% | 59.90% | 74.28% | 36.79% | 60.07% | 63.98% | 29.98% | 54.58% | 72.40% | 38.66% | 48.90% | 42.13% |
